# Overwintering temperature and body condition shift emergence dates of spring-emerging solitary bees

**DOI:** 10.7717/peerj.4721

**Published:** 2018-05-16

**Authors:** Mariela Schenk, Oliver Mitesser, Thomas Hovestadt, Andrea Holzschuh

**Affiliations:** Department of Animal Ecology and Tropical Biology, University of Würzburg, Würzburg, Germany

**Keywords:** Wild bees, Timing, Fitness, Hibernation, Climate change, Mechanistic model, *Osmia*, Body weight, Body size, Pollinators

## Abstract

Solitary bees in seasonal environments must align their life-cycles with favorable environmental conditions and resources; the timing of their emergence is highly fitness relevant. In several bee species, overwintering temperature influences both emergence date and body weight at emergence. High variability in emergence dates among specimens overwintering at the same temperatures suggests that the timing of emergence also depends on individual body conditions. However, possible causes for this variability, such as individual differences in body size or weight, have been rarely studied. In a climate chamber experiment using two spring-emerging mason bees (*Osmia cornuta* and *O. bicornis*), we investigated the relationship between temperature, emergence date, body weight, and body size, the last of which is not affected by overwintering temperature. Our study showed that body weight declined during hibernation more strongly in warm than in cold overwintering temperatures. Although bees emerged earlier in warm than in cold overwintering temperatures, at the time of emergence, bees in warm overwintering temperatures had lower body weights than bees in cold overwintering temperatures (exception of male *O. cornuta*). Among specimens that experienced the same overwintering temperatures, small and light bees emerged later than their larger and heavier conspecifics. Using a simple mechanistic model we demonstrated that spring-emerging solitary bees use a strategic approach and emerge at a date that is most promising for their individual fitness expectations. Our results suggest that warmer overwintering temperatures reduce bee fitness by causing a decrease in body weight at emergence. We showed furthermore that in order to adjust their emergence dates, bees use not only temperature but also their individual body condition as triggers. This may explain differing responses to climate warming within and among bee populations and may have consequences for bee-plant interactions as well as for the persistence of bee populations under climate change.

## Introduction

In seasonal environments, climate has a powerful influence on the timing of many spring events such as plant flowering, breeding of birds, and the arrival of migrant species (Walther et al., 2002; Parmesan & Yohe, 2003; Gordo & Sanz, 2010; Aldridge et al., 2011). As life-cycles of organisms must align with favorable environmental conditions and resources (Van Asch & Visser, 2007; Donoso et al., 2016), proper timing of phenological events is highly fitness relevant (Bradshaw & Holzapfel, 2007). Pollinating insects such as solitary bees, for example, have to time their emergence so that their activity period matches the phenology of their food plants (and vice versa). As spring progresses, mean ambient temperature and availability of flower resources increase (Schwartz & Karl, 1990), leading to increasingly favorable emergence conditions for spring-emerging solitary bees. However, waiting for the most favorable environmental conditions may not be the best strategy, as waiting carries an opportunity cost. Waiting too long may needlessly shorten the overall time available for reproduction. As the season progresses the number of emerged bees increases, and also if a large number of bees emerge simultaneously, intra-specific competition for mating partners (in the case of males) and inter- and intra-specific competition for nesting sites (in the case of females) are increasing. Individuals emerging earlier may thus gain fitness benefits (Poethke, Hovestadt & Mitesser, 2016). Choosing the right moment for emergence may therefore require balancing these different risks and benefits.

Temperature is generally regarded as having a strong influence on the timing of bee emergence in temperate systems; statistical modelling approaches have previously shown that the emergence dates of solitary bees can be explained by including temperature-related factors such as lower temperature thresholds and degree days requirements (White, Son & Park, 2009; Forrest & Thomson, 2011). However, solitary bees show considerable variability in emergence dates even if they overwinter at the same location with the same overwintering temperature (Westrich, 2011). To our knowledge, the mechanisms and causes underlying this variation cannot be explained with certainty, but the speculation is a (maternal) bet-hedging strategy, which can be expected to pay off in environments with unpredictable environmental variability (Danforth, 1999; Hopper, 1999; Childs, Metcalf & Rees, 2010; Poethke, Hovestadt & Mitesser, 2016). Our study tries to clarify the ultimate and proximate causes of this high variability in emergence dates of solitary bees.

It has been shown in various species that life-history strategies can be dependent on individual conditions, especially on body size. For example, natal movement in juvenile Atlantic salmon represents a body size-dependent strategy with larger individuals staying closer to the nest than their smaller conspecifics (Einum et al., 2012). Another example is the red fox: larger males are more present at boundaries than their lighter conspecifics and therefore have larger territories (Iossa et al., 2008). In bees, larger individuals of the same species are able to forage and to collect pollen when ambient temperatures are low (Stone, 1993; Stone, 1994). Large and heavy bees may also have a higher probability of surviving starving periods after emergence, during which insects in general rely on fat reserves (Arrese & Soulages, 2010, Weissel et al., 2012), because they may have greater fat reserves than their smaller conspecifics. In addition, large individuals have more offspring than their smaller conspecifics, which makes body size (or body weight) a key component of fitness in solitary bees (Larsson, 1990; Kim, 1997). Hence, we expect that within a population of spring-emerging bees, larger individuals emerge earlier to exploit potential benefits of early emergence because they are better able than their smaller conspecifics to cope with the risks of harsh weather conditions and low food availability associated with early emergence. We thus assume that solitary bees consider not only environmental factors such as overwintering temperature but also their own body condition for adjusting their emergence dates. As overwintering temperature also has an influence on the loss of body weight during winter (and therefore on the fitness) of spring-emerging solitary bees (Fliszkiewicz et al., 2012; Fründ, Zieger & Tscharntke, 2013), we want to disentangle the relationship between body weight and body size, overwintering temperature, and emergence date of solitary bees. This will enable us to more accurately assess the impacts of climate warming on the timing and the fitness of solitary bees, and to provide more precise predictions about the persistence of these species.

To evaluate these issues, we performed an experiment in which we focused on the weight loss of bees during hibernation, on their emergence dates, and on their body size and weight at time of emergence under average, above average, and below average overwintering temperatures in three climate chambers. We focused on two solitary bee species of the genus *Osmia* that emerge in early and in mid-spring, respectively. The following questions were addressed: (1) Is the slope of body-weight decline during overwintering affected by temperature (cold, medium and warm overwintering), and are bees that overwinter under warm temperatures lighter at the time of emergence than bees that overwinter under medium or cold temperatures? (2) How do overwintering temperatures (cold vs. medium vs. warm) influence emergence dates? (3) How does body size (or body weight at the time of emergence) influence emergence dates within a population that experiences the same overwintering temperatures? In addition, we developed a simple mechanistic model that allows us to unite our observations into a consistent framework, and thus to explain the (potential) adaptive benefits of different responses to the environment.

## Materials and Methods

### Bees

We focused on two spring-emerging solitary bee species (Hymenoptera: Apiformes: Megachilidae): the hornfaced mason bee *Osmia cornuta* has an activity period from March until May, whereas the red mason bee *Osmia bicornis* has an activity period from early April until June (Westrich, 2011). Like most solitary bee species that emerge in early spring, *O. cornuta* and *O. bicornis* overwinter in their cocoons as fully developed adults that remain inside their brood cells; they emerge in spring when temperatures rise (Bosch & Kemp, 2002). The initial body weight of a bee (and therefore its initial amount of internal fat reserves) is solely determined by the amount of pollen stocked by the mother inside its brood cell in the previous season (and which the larva fully consumes before pupating) (Bosch & Vicens, 2002). During the overwintering period, *O. cornuta* and *O. bicornis* are no longer provided with food (Westrich, 2011) and thus have to live from their internal fat reserves. Male bees in general emerge some days if not weeks before the females of the same species (Raw, 1972; Westrich, 2011). Cocoons of both species were purchased from “WAB Mauerbienenzucht“ (Konstanz, Germany), a commercial supplier of solitary bees. From October 2013 until the start of the experiment in December 2013, cocoons were stored inside a climate chamber at constant 4 °C.

### Experimental design

In three climate chambers (Panasonic Cooled Incubator MIR-254-PE; Panasonic, Kadoma, Japan), we established three overwintering temperature treatments based on long term (65 years) daily means obtained from the regional climate station in Würzburg, Germany (DWD Climate Data Center CDC 2016). For each month between December and June we calculated the monthly mean temperatures; these were then used to regulate standard temperatures in the climate chambers. On the basis of these values, we implemented the following temperature treatments: (1) warm overwintering temperature (=monthly mean + 3 °C), (2) medium overwintering temperature (=monthly mean) and (3) cold overwintering temperature (=monthly mean − 3 °C). Temperatures were shifted monthly in all treatments, but were kept constant within months (Table A1). We defined each month as 30 days in duration. The experiment started on the 1st of December 2013 and lasted until the last bee emerged the following spring (11th June 2014). To control the accuracy of climate chambers, temperature and humidity inside the chambers were recorded every 20 min. For this purpose, sensors (Driesen & Kern DK390 ECH20 HumiLog GP “rugged”; Driesen Kern GmbH, Bad Bramstedt, Germany) were located in the center of each chamber.

Into each climate chamber, 600 cocoons per bee species were placed. The cocoons (3,600 in total) were individually put into ID-labelled plastic tubes that were sealed with cotton wool (Fig. A1).

### Data recording

Half the cocoons were used to assess changes in bees’ dry weights during overwintering (300 cocoons × 3 chambers × 2 species = 1,800 cocoons in total). On the starting date of the experiment and on the last day of each month (every 30 days), we removed 22 cocoons (11 females and 11 males) per species and treatment. Cocoons were randomly and successively removed from the experiment and opened until we had collected 11 male and 11 female bees; superfluous bees (>22) were dismissed. Directly after removal, bees were killed in a freezer at −80 °C. Dry weight was determined after drying specimens for 48 h at 60 °C by weighing dried specimens within 10 min after taking them from the drying oven to avoid moisture absorption. We then measured the head width of specimens, as this measure has previously been shown to be a reliable correlate of body size (Bosch & Vicens, 2002; O’Neill, Delphia & O’Neill, 2014). In all analyses, we used dry weight corrected for body size (dry weight / head width^2^) as an estimate of the fat reserves of the bees. Hereafter we refer to this variable as body weight.

The remaining cocoons were left inside the chambers until emergence. These bees were used to assess emergence dates and emergence weights. Starting daily in February, we checked and recorded the emergence of bees; emerged bees were removed from climate chambers, killed and dried to determine their body weight as described above. From the initial remaining 300 cocoons per species and treatment, 240–290 bees ultimately emerged (Table A2).

### Statistical analyses

For statistical analysis of the data we used software R (R Core Team, 2018). Separate models were fitted for each bee species. To test whether body weight declined during overwintering and whether the slope of the decline differed among temperature treatments, we used a general linear model with temperature treatment (medium vs. warm vs. cold overwintering temperature), sex, date and all their interactions as predictors and with body weight measured monthly as response variable. For each monthly body weight measurement new specimens were used (see above). Non-significant predictors (*p* > 0.05) were removed from the model in a manual stepwise model selection (Crawley, 2007). To detect differences in body weight at time of emergence among temperature treatments, we used separate general linear models for males and females with body weight as response variable and temperature treatment as predictor. Temperature treatments were compared using treatment contrasts (Crawley, 2007). To assess how overwintering temperature and body size (or body weight at time of emergence) affected emergence date, we used two general linear models with emergence date as response variable, and treatment, sex and body size (or body weight), and all their interactions as predictors. Non-significant predictors (*p* > 0.05) were removed from the models in a manual stepwise model selection (Crawley, 2007). Model residuals were inspected for violation of assumptions or normality and homoscedasticity.

## Results

**Figure 1:**
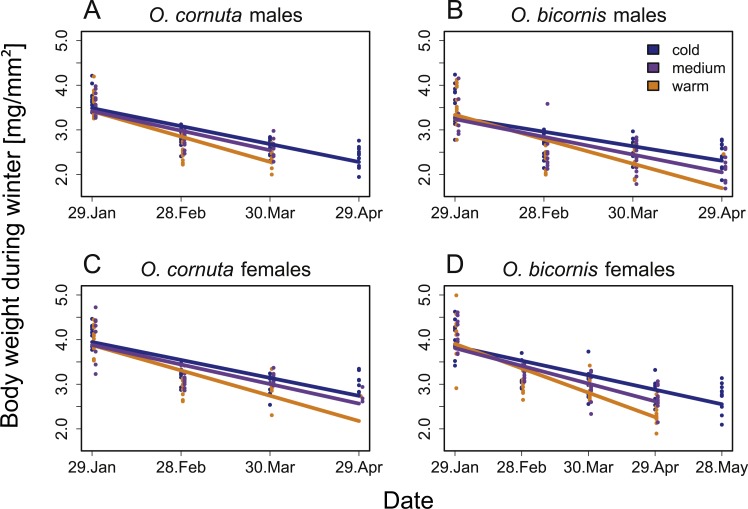
Influence of date on the body weight during overwintering of (A) *O. cornuta* males, (B) *O. bicornis* males, (C) *O. cornuta* females and (D) *O. bicornis* females. Measurements at each date were taken from a new group of 22 bees per temperature treatment (orange: warm, purple: medium, blue: cold overwintering temperature treatment). Body weight was corrected for body size [mg/mm^2^]. Points show the raw data. Regression lines represent the results of the general linear model.

The body weights of *O. cornuta* and *O. bicornis*, both male and female, decreased over time during overwintering. A significant interaction between temperature treatment and date indicated that the slope of this relationship depended on temperature treatment (warm vs. medium vs. cold): The body weight of both species and sexes decreased most strongly under the warm temperature and least under the cold temperature, with the medium temperature in between (Table 1, Fig. 1).

**Table 1:** Effects of temperature, sex and date on the body weight of *O. cornuta* and *O. bicornis*. We provided three different temperature treatments (warm vs. medium vs. cold overwintering temperature treatment). Body weight was corrected for body size [mg/mm^**2**^]. Non-significant predictors (*p* > 0.05) were removed from the model in a manual stepwise model selection.

	***O. cornuta***				***O. bicornis***		
	*Df*	*F*	*p*		*Df*	*F*	*p*
**Body weight during winter [mg/mm**^**2**^**]**				**Body weight during winter [mg/mm**^**2**^**]**			
Temperature	2	2.33	0.099	Temperature	2	1.66	0.19
Sex	1	98.87	<0.001	Sex	1	87.53	<0.001
Date	1	414.96	<0.001	Date	1	397.40	<0.001
Temperature : Date	2	4.46	0.013	Temperature : Date	2	9.74	<0.001

**Figure 2:**
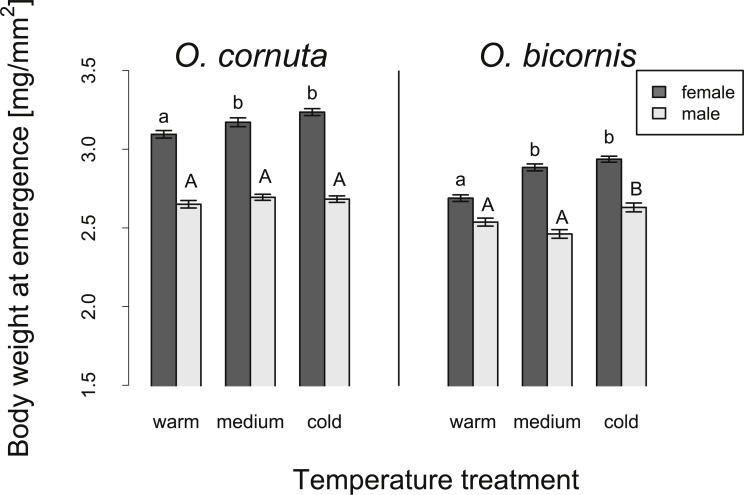
Influence of temperature on the body weight at emergence of *O. cornuta* and *O. bicornis* males and females. We compared three different temperature treatments (warm, medium and cold overwintering temperature treatment). Body weight was corrected for body size [mg/mm^2^]. Different letters above bars (means ± SE) indicate significant differences among temperature treatments (*p* < 0.05).

At emergence, females of *O. cornuta* and males and females of *O. bicornis* had reduced body weights under the warm temperature treatment as compared to the cold temperature treatment; females of both species also had a reduced body weight at emergence in the warm temperature treatment as compared to the medium temperature treatment. The body weights of male *O. cornuta* were not significantly influenced by temperature treatment (Table 2, Fig. 2).

**Table 2:** Results of linear models testing differences in body weights at emergence among temperature treatments. Shown are treatment contrasts between warm, medium and cold overwintering temperatures. Dependent variable is body weight corrected for body size [mg/mm^**2**^] of *O. cornuta* and *O. bicornis* at the time of emergence. *P*-values in bold indicate significant results (*p* < 0.05).

	***O. cornuta*** **males**			***O. cornuta*** **females**			***O. bicornis*** **males**			***O. bicornis*** **females**		
	*Df*	*t*	*p*	*Df*	*t*	*p*	*Df*	*t*	*p*	*Df*	*t*	*p*
**Body weight at emergence [mg/mm**^**2**^**]**												
warm vs. medium	2	1.51	0.132	2	2.16	**0.031**	2	−1.92	0.056	2	−6.61	**<0.001**
warm vs. cold	2	1.03	0.302	2	4.19	**<0.001**	2	2.52	**0.012**	2	8.16	**<0.001**
medium vs. cold	2	−0.39	0.700	2	1.82	0.069	2	4.28	**<0.001**	2	1.81	0.071

**Figure 3:**
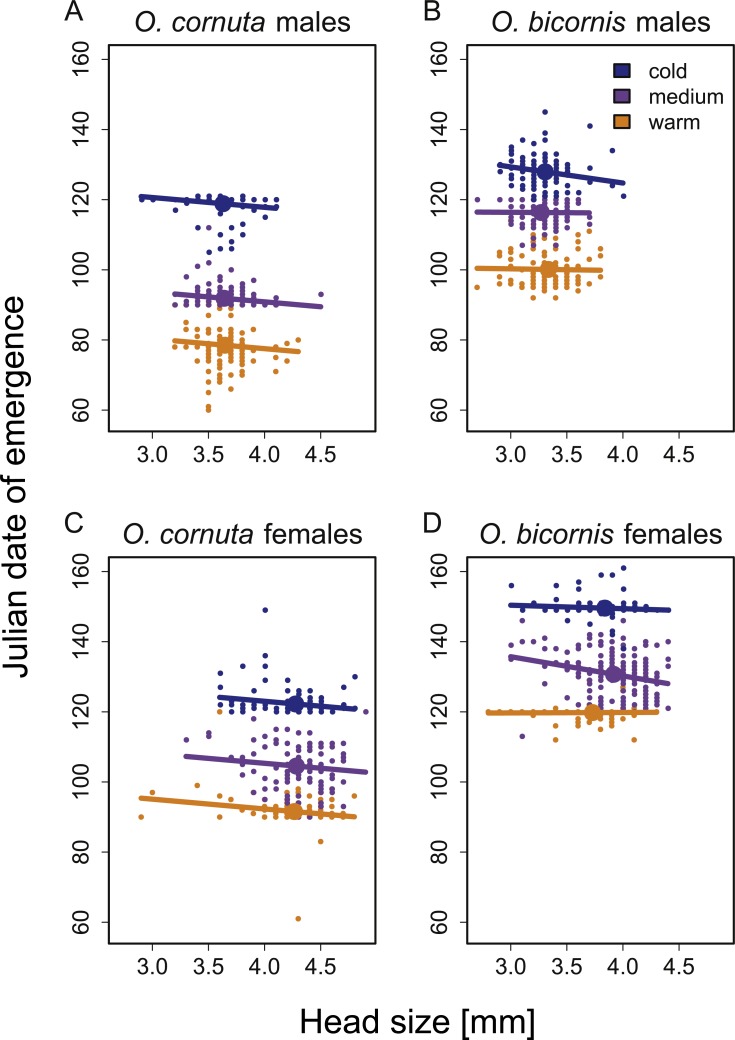
Influence of temperature and head size on the Julian date of emergence of (A) *O. cornuta* males, (B) *O. bicornis* males, (C) *O. cornuta* females and (D) *O. bicornis* females. We compared three different temperature treatments (orange: warm, purple: medium, blue: cold overwintering temperature treatment). Small points show the raw data. Regression lines represent the results of general linear models. Bold points show the mean head size and the mean Julian date of emergence of each temperature treatment. Julian date 100 = 10th of April.

Temperature treatment (warm vs. medium vs. cold) had an influence on the emergence dates of *O. cornuta* and *O. bicornis*. Males and females of both species emerged earlier under warm and later under cold treatments as compared to the medium temperature treatment (Table 3, Fig. 3).

**Table 3:** Effects of temperature, sex and head size or body weight on the Julian date of emergence of *O. cornuta* and *O. bicornis*. We compared three different temperature treatments (warm vs. medium vs. cold overwintering temperature treatment). Body weight was corrected for body size [mg/mm^**2**^]. Non-significant predictors (*p* > 0.05) were removed from the model in a manual stepwise model selection. Julian date 100 = 10th of April.

	***O. cornuta***				***O. bicornis***		
	*Df*	*F*	*p*		*Df*	*F*	*p*
**Julian date of emergence**				**Julian date of emergence**			
Temperature	2	3,641.46	<0.001	Temperature	2	3,499.88	<0.001
Sex	1	794.33	<0.001	Sex	1	3,977.72	<0.001
Head size	1	16.83	<0.001	Head size	1	26.70	<0.001
Temperature : Sex	2	80.84	<0.001	Temperature : Sex	2	47.11	<0.001
				Temperature : Head size	2	5.98	0.003
				Sex : Head size	1	0.01	0.921
				Temperature : Sex : Head size	2	3.15	0.043
**Julian date of emergence**				**Julian date of emergence**			
Temperature	2	3,868.56	<0.001	Temperature	2	3,734.16	<0.001
Sex	1	843.87	<0.001	Sex	1	4,243.99	<0.001
Body weight	1	74.83	<0.001	Body weight	1	76.39	<0.001
Temperature : Sex	2	77.91	<0.001	Temperature : Sex	2	46.95	<0.001
Temperature : Body weight	2	3.60	0.028	Temperature : Body weight	2	8.39	<0.001
				Sex : Body weight	1	0.01	0.913
				Temperature: Sex : Body weight	2	8.84	<0.001

Emergence date and head size (which is not affected by overwintering temperatures) were negatively correlated for both species and sexes: individuals with large head size emerged earlier than individuals with small head size (Table 3, Fig. 3). In *O. bicornis*, the significant interaction between temperature treatment, sex and head size demonstrated that the slope of this relationship depended on temperature treatment (warm vs. medium vs. cold) and sex, though without a clear pattern among temperature treatments. Emergence date and body weight at emergence were also negatively correlated for both species and sexes. In all temperature treatments (warm, medium and cold), individuals with high body weight emerged before individuals with low body weight (Table 3, Fig. A2). The significant interaction between temperature treatment and body weight in both species indicated that the slope of this relationship depended on temperature treatment (warm vs. medium vs. cold), though without a clear pattern among temperature treatments. Although body weight and head size were overall highly significantly correlated to the emergence date of bees (Table 3), we occasionally observed ‘mass-emergence events’ where many bees with differing body weights emerged on the same day (e.g., on day 120 86 of 113 *O. cornuta* males emerged in the cold temperature treatment and 120 of 156 *O. bicornis* females emerged in the warm temperature treatment).

## Discussion

We demonstrated that spring-emerging solitary bees lost body weight during hibernation and that the slope of decline over time was steeper in warm than in cold overwintering temperatures. These results are in accordance with previous studies on solitary bees, which have suggested negative effects of increased overwintering temperatures on the body weight and fat body mass of bees (Fliszkiewicz et al., 2012; Fründ, Zieger & Tscharntke, 2013; Wasielewski et al., 2013). In warm temperatures metabolic functions are more rapid and overall energy expenditure is thus higher than in cold temperatures (Vesterlund & Sorvari, 2014). Further, at the time of emergence, bees overwintering in warm temperatures had, despite emerging about a month earlier than bees in cold temperatures, lower body weight than bees overwintering in medium (females of both species) or cold temperatures (all except male *O. cornuta*). This indicates that the fat reserves saved by shortening the overwintering period did not fully compensate for the higher monthly weight loss in warm temperatures. As fitness depends on body weight (or body size) in solitary bees (Larsson, 1990; Kim, 1997; Seidelmann, Ulbrich & Mielenz, 2010), we assume that bees in warm overwintering temperatures not only emerge earlier with a decreased body weight but also have lower fitness expectations than bees kept in cold overwintering temperatures. There is evidence that solitary bees show signs of decreased survival and decreased longevity when overwintered in warm temperatures (Bosch & Kemp, 2003), but future studies should investigate how temperature affects fat reserves of the bees and their fitness measured as the number of offspring.

In agreement with previous studies (Bosch & Kemp, 2000; Bosch & Kemp, 2003; Bosch & Kemp, 2004; White, Son & Park, 2009; Fründ, Zieger & Tscharntke, 2013), both bee species emerged earlier under warm than cold overwintering temperatures. Our study further showed that under all temperature treatments, emergence date was negatively correlated to body weight for both species and sexes and to body size (though the latter is unaffected by overwintering temperatures). Among specimens that experienced the same overwintering temperatures, small and light bees emerged later than their larger and heavier conspecifics. We are aware of only a single other study which finds, for a desert bee species, a relationship between body weight and emergence date (Danforth, 1999). However, the trend reported in that study contrasted with our findings: after onset of the annual rainfall period, which marks the start of food plant flowering, low body weight individuals were more likely to emerge than those with high body weight. The portion of the population, characterized by high average body weight remained in diapause for another year. This was interpreted as a bet-hedging strategy in which only the heaviest individuals have enough energy resources for postponing emergence for one year (Danforth, 1999). Our study, however, focused on the variation in emergence dates within one spring season. In temperate climates, larger individuals from the same species have been shown to successfully forage at cooler temperatures (Stone, 1993; Stone, 1994) and they may also have greater fat reserves than smaller individuals. This would enable them to survive under periods of starvation outside the nest. Therefore, we conclude that within a population of spring-emerging solitary bees, larger individuals emerge earlier in order to seek potential benefits of early emergence as they may be better able to cope with the harsher weather conditions and low food availability that occur early in spring. This effect of body size on the emergence dates of bees seems to be especially strong for bee species emerging in very early spring when the risk of adverse weather conditions is particularly high: we found a consistent effect of body size on the emergence dates in *O. cornuta*, while the effect was less consistent in the later emerging *O. bicornis,* which had different slopes in the body size emergence date relationships among temperature treatments and sexes.

**Figure 4:**
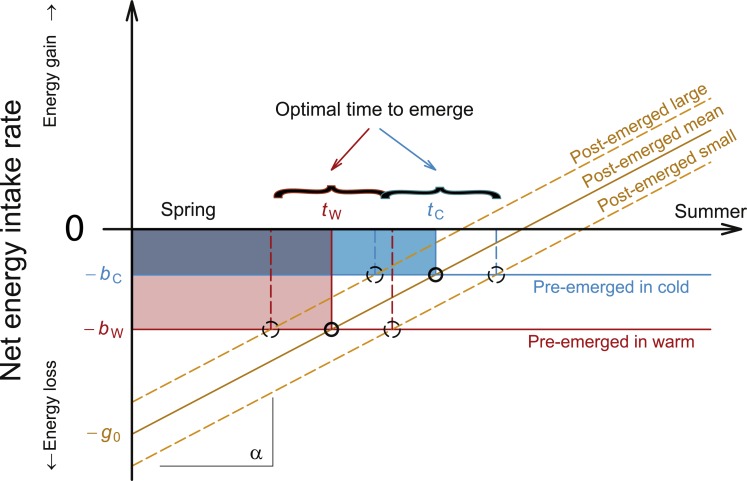
Schematic presentation of mechanistic model predicting the optimum emergence date for bee individuals with different body condition and under different overwintering temperatures. –*b*_*c*_: net energy intake rate for inactive (pre-emerged) bee in cold overwintering temperature. –*b*_*w*_: net energy intake rate for an inactive (pre-emerged) bee in warm overwintering temperature. –*g*_0_: net energy gain for an active (post-emerged) bee from natural resources. *α*: (daily) increase in net energy intake rate for an active (post-emerged) bee in spring. *t*_*w*_: optimal time to emerge for a bee in warm overwintering temperatures. *t*_*c*_: optimal time to emerge for a bee in cold overwintering temperatures. The red area represents the cumulative (integrated) weight loss for bees under warm and the blue area represents the cumulative weight loss for bees under cold overwintering temperatures. As long as −*g*_0_ is not close to –*b*_*w*_ (very short winter) the red area will be larger than the blue, indicating higher energy consumption of bees during warmer but briefer overwintering periods than of bees overwintering under cold conditions. In Appendix 2 we provide a more formalized and precise version of this argument.

To further develop our arguments, we present in the following a simple mechanistic model that provides a coherent and strategic explanation for our different observations by predicting the optimum emergence dates for bee individuals with different body conditions and under different overwintering temperatures. The model is explained step by step with a graphical presentation provided in Fig. 4; in Appendix 2 we provide an analytical formulation of the model. The model is based on our results and on data from the literature which show that during spring, the availability of blossoms and consequently the possible net energy intake rate of post-emerged bees slowly increase. The model builds on the assumption that bees take a strategic decision on when to emerge based on balancing fitness expectations associated with either remaining in the cocoon or emerging and becoming active. We first integrated the observed greater weight loss by bees in warm, rather than in cold, overwintering temperatures into our model via the more negative net energy intake rate of pre-emerged bees in warm overwintering temperatures than that of pre-emerged bees in cold overwintering temperatures (indicated by the red and the blue horizontal lines in Fig. 4). If food resources outside the nest are scarce or absent, a post-emerged, active bee would certainly lose more energy than a pre-emerged, inactive bee in its cocoon. The availability of flower resources increases with the onset of spring (Schwartz & Karl, 1990) and likely also the probability of successful foraging trips for bees. Therefore, the expected net energy intake rate of post-emerged bees should gradually increase during spring (an effect indicated by the orange upward sloping line in Fig. 4). At some point the negative net energy intake rates of pre-emerged bees in warm and cold overwintering temperatures will intersect (though at different points in time) with this orange line; a bee should emerge when its (expected) net energy intake rate becomes larger outside than inside the cocoon. In Fig. 4, these optimal moments of emergence are in each case marked by the black-circled intersection point of lines; our model indicates an earlier optimal emergence date for bees kept under warm compared to those kept under cold overwintering temperatures, which is consistent with our empirical results. In fact, this effect would even be enhanced if warmer temperatures (at the same time) also indicated an earlier availability of flowers (which would shift the line for the potential net energy intake rate of post-emerged bees to the left). We also conclude that –as long as the overwintering period is not very short- the weight loss of bees until emergence should typically be greater in warm than in cold overwintering conditions. This is in agreement with the empirical findings of this study and indicated in Fig. 4 by the differently-sized and differently-colored areas: The larger red area represents the cumulative (integrated) weight loss for bees in warm and the smaller blue area for bees in cold overwintering temperatures; for further explanations of this topic see Appendix 2. To account for the greater foraging efficiency of large-sized bees (Seidelmann, Ulbrich & Mielenz, 2010), we adjusted potential net energy intake rates accordingly: the orange dotted line representing net energy intake rate for large-sized bees falls above that for small-sized post-emerged bees (Fig. 4). Consequently, the model predicts that the optimal date of emergence occurs earlier for large-sized bees than for small-sized bees under identical environmental conditions, as large-sized bees benefit from emerging at an earlier time point (compare black dotted circled intersection point of lines of large- and small-sized bees, Fig. 4). We consistently observed earlier emergence of large-sized rather than small-sized bees. It has not escaped our notice that under certain circumstances ‘mass emergence events’ occurred, with many bees of differing body weights emerging on the same day (Fig. A2). Our model is also capable of explaining the reasons for these ‘mass emergence events’ and the reasons why these events do not always occur after an abrupt change in temperature (for more details see Fig. A3).

Our mechanistic model thus explains all our observations by a consistent theoretical scheme. In combination with the empirical data, the model strongly suggests that solitary bees follow a strategic approach and emerge at a date that is most profitable for their individual fitness expectations. An alternative strategy is also conceivable, namely that bees emerge when their body weight falls below a critical value. If this were true, however, bees would emerge either at exactly the same body weights, or lighter bees would emerge before their heavier conspecifics without falling below the critical body weight. However, we showed that heavier bees emerge before their lighter conspecifics. Therefore, we conclude that solitary bees do not emerge earlier in warm overwintering temperatures because they cannot afford to wait longer due to increased weight loss. Instead, solitary bees in warm overwintering temperatures emerge earlier because they benefit at an earlier date from the emergence event than bees in cold overwintering temperatures. Among specimens experiencing the same overwintering temperatures, larger individuals tended to emerge earlier than their smaller conspecifics because they benefited at an earlier date from the emergence event than their smaller conspecifics.

Our finding that large bees emerge earlier than small bees can improve predictions on how bee populations will be affected if climate change desynchronizes plant–pollinator interactions. For example, if under climate warming bees advance their phenologies more strongly than plants (Willmer, 2014), our results suggest that, in particular, large-sized bee individuals will be desynchronized with their food plants because they emerge earlier than their smaller conspecifics. Bees emerging only six days before the onset of their food plants’ flowering fail to produce offspring (Schenk, Krauss & Holzschuh, 2018). An increased risk of desynchronization for large individuals, which are expected to have higher reproductive output than small individuals (Larsson, 1990; Kim, 1997), may enhance the negative effects of desynchronization on bee populations. An alternative scenario is that, in response to climate warming, bees and plants show equivalent shifts in their phenologies (Bartomeus et al., 2011) or that plants advance their phenologies even more rapidly than bees (Forrest & Thomson, 2011; Kudo & Ida, 2013). If this scenario were accompanied by resource scarcity, it would lead to reduced body sizes (or body weights) in the bee progeny (Bosch & Vicens, 2002). Possible causes of resource scarcity and thus reduced pollen resources are habitat loss (Clough et al., 2014) or changes in precipitation patterns (Rafferty, Bertelsen & Bronstein, 2016). A lack of nest cavities leads to utilization of suboptimal smaller nesting tubes which then also results in smaller offspring (Seidelmann, Bienasch & Prohl, 2016). As small-sized bees emerge later than their larger conspecifics, emergence dates of bees could then be delayed which in turn may increase the danger that bees cannot keep pace with phenology shifts in plants leading to a decrease in plant pollination. However, the consequences of global warming to bee emergence and plant–pollinator interactions remain difficult to predict given the lack of knowledge on how phenotypic plasticity and variability among populations affect the genetic architecture of pollinator populations in space and time.

## Conclusion

Our empirical data and our mechanistic model clearly suggest that bees emerge at times that maximize their (expected) fitness. We have shown that these dates are, on the one hand, temperature dependent; warmer overwintering temperatures increase the weight loss of bees during hibernation, which then advances their optimal emergence date to an earlier time point (due to an earlier benefit from the emergence event). On the other hand, our findings also suggest that the optimal emergence date depends on individual body size (or body weight) as bees adjust their emergence date according to their foraging ability and possibly their ability to cope with harsh conditions early in the season. We therefore suggest that it is not enough solely to investigate temperature effects on the timing of bee emergence, but to also consider individual body conditions of solitary bees in order to understand the timing of bee emergence. Only then we will be able to give more precise predictions about the risks and consequences of temporal mismatches between bees and food plants and the persistence of these bee species under changing environmental conditions.

##  Supplemental Information

10.7717/peerj.4721/supp-1 Appendix 1 Additional figures, tables and information

10.7717/peerj.4721/supp-2 Appendix 2 Analytical formulation of the model

10.7717/peerj.4721/supp-3 Supplemental Information 1 BMI during winter

10.7717/peerj.4721/supp-4 Supplemental Information 2 Emergence dates and BMI at emergence

## Additional Information and Declarations

### Data Availability

The following information was supplied regarding data availability: The raw datasets are provided as Supplemental Files.
