# Supplementary material for: Overwintering temperature and body condition shift emergence dates of spring-emerging solitary bees
Source: PeerJ. 2018 May 16;6:e4721. doi: 10.7717/peerj.4721 (PMC5960268; doi:10.7717/peerj.4721)
Supplement: Appendix 1 [file peerj-06-4721-s001.docx]

**Appendix 1**

**Table A1** Monthly values of the four temperature treatments. Temperature treatments were based on long-term (65 years) daily means from the regional climate station in Würzburg, Germany: constant warm (=mean + 3°C), constant medium (=mean) and constant cold (=mean - 3°C). Temperatures changed monthly in all treatments, but were constant within months.

**
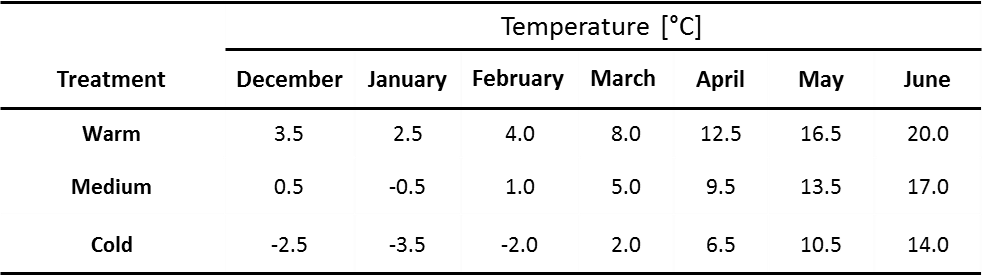
**

**Table A2** Number of emerged females and males of *O.cornuta* and *O.bicornis* from each temperature treatment.


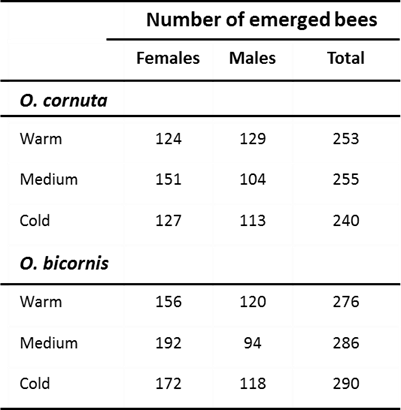


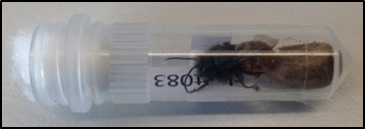


**Fig. A1** Cocoons were stored inside plastic tubes that were sealed with cotton wool. Female *Osmia cornuta* that left its cocoon (=emerged).


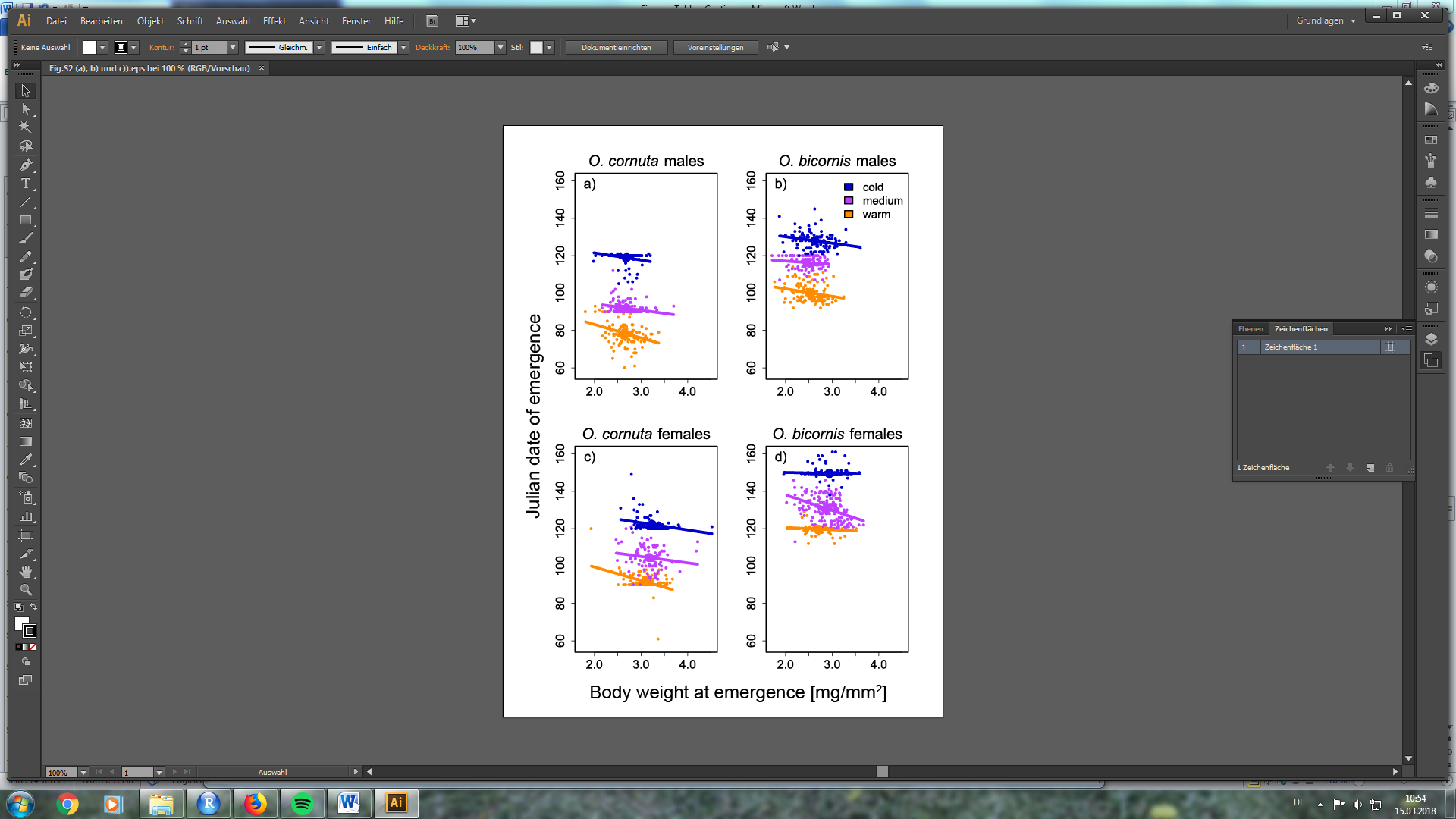


**Fig. A2** Influence of temperature and body weight on the Julian date of emergence of a) *O. cornuta* males, b) *O. bicornis* males, c) *O.cornuta* females and d) *O.bicornis* females. We compared three different temperature treatments (orange: warm, purple: medium, blue: cold overwintering temperature treatment). Body weight was corrected for body size [mg/mm^2^]. Small points show the raw data and regression lines represent the results of general linear models. Bold points show the mean body weight and the mean Julian date of emergence of each temperature treatment. Julian date 100 ≙ 10^th^ of April.


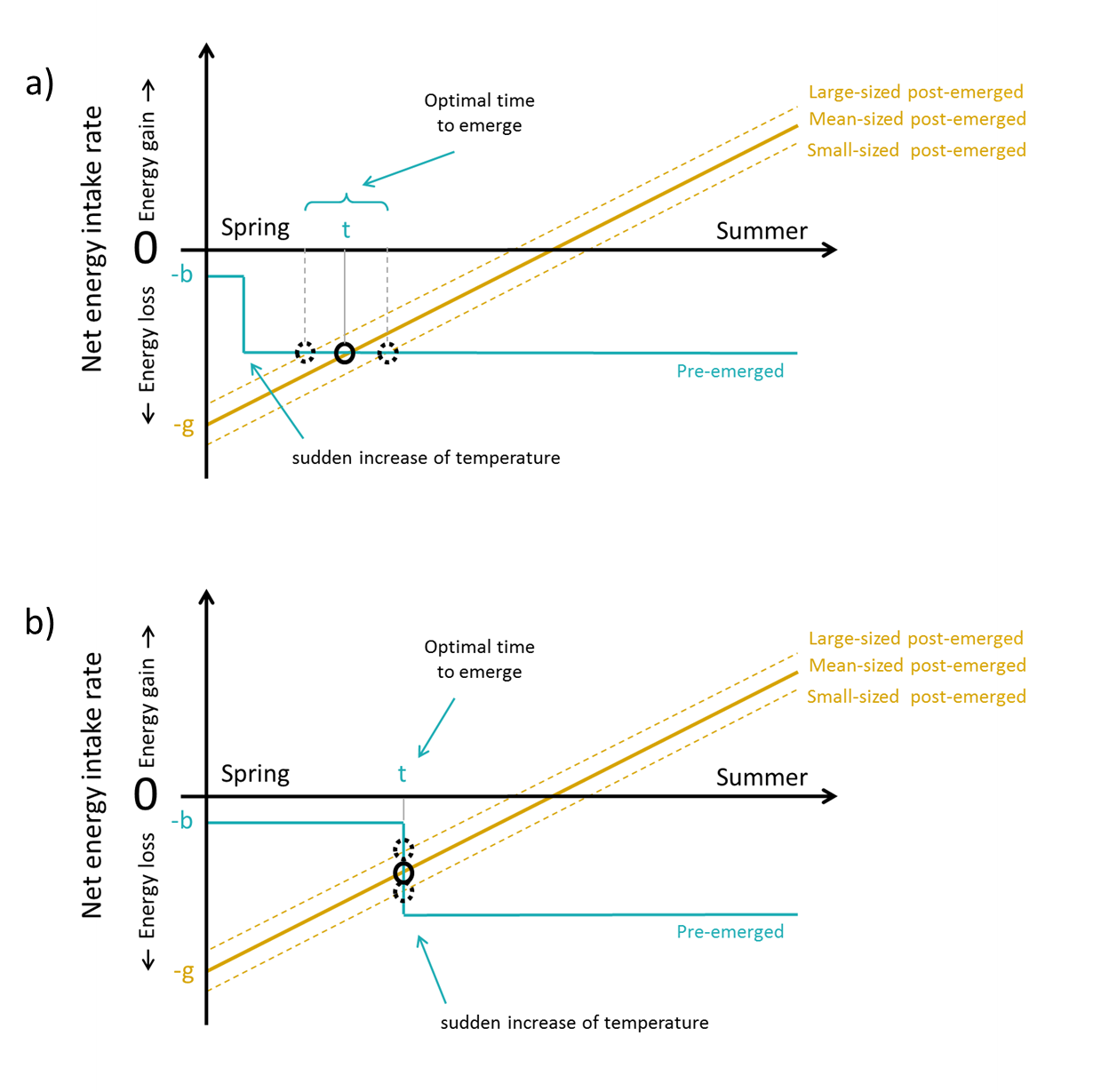


**Fig. A3** Mechanistic model predicting the optimum emergence date for bee individuals under a sudden increase in temperature (a) well before bees would start to emerge and (b) immediately before bees start to emerge. -b: net energy intake rate for an inactive (pre‐emerged) bee. -g: net energy gain for an active (post‐emerged) bee from natural resources. t: optimal time to emerge.

**Explanation:** It has not escaped our notice that under certain circumstances ‘mass emergence events’ occurred with many bees of differing body sizes and body weights emerging on the same day (Fig. 2 and Fig. A2). As such events were exclusively observed on day 90 and on day 120 after the start of the experiment, we attribute them to the triggering effect of the monthly temperature adjustment (which we performed every 30 days: at day 0, 30, 90, 120 and at day 150 after the start of the experiment). Our model is capable of explaining both the reason for the occurrence of such ‘mass emergence events’ and also the reason why such events do not occur always after an abrupt change in temperature (this further underscores the credibility of our model): In the case of a sudden temperature increase (which increases the energy loss of bees during overwintering) well before bees would start to emerge (a), the temperature increase has no effect on the natural variability in emergence dates of bees (large-sized bees emerge before small-sized bees). However, in the case of a sudden temperature increase immediately before bees start to emerge (b), many bees with differing body sizes emerge on the same date.

Certainly, the amount of inter- or intraspecific competition for nesting sites and the current quantity of available nesting sites or mating partners may also play a role in determining the most profitable emergence date of a bee and our mechanistic model could indeed be extended to account for such additional factors. Nevertheless, even in its current form our model is able to explain all our results, signaling that we included the most important factors affecting the timing of emergence -namely ambient winter temperature and body size (or body weight).
