# Supplementary material for: Overwintering temperature and body condition shift emergence dates of spring-emerging solitary bees
Source: PeerJ. 2018 May 16;6:e4721. doi: 10.7717/peerj.4721 (PMC5960268; doi:10.7717/peerj.4721)
Supplement: Appendix 2 [file peerj-06-4721-s002.docx]

**Appendix 2**

In the following we provide a very simple but mechanistically based model that is built only on three assumptions: (1.) that metabolic rate and thus fat consumption depends on environmental temperature and is higher under warm than under cool conditions, (2.) that in spring the (expected) net-energy intake rate of an active bee would increase as the season progresses, and (3.) that larger body size improves foraging performance (under cool temperatures) and thus net-energy intake rate. Assumptions (1.) and (3.) had already been tested independently, as explained in the main text. In seasonal habitats, the second assumption can be assumed to be true.

Based on these assumptions we constructed a model that is capable of unifying and explaining the different empirical observations. Note that in principle, the passage of time should be measured from the beginning of hibernation viz. the end of maternal food provisioning in last season. However, as we are interested in explaining the **differences** in energy consumption and timing of emergence as sparked by the experimental treatment, we consider only the winter phase during which individuals are exposed to different temperature conditions; before setting up the experiments all bee cocoons were kept under the same conditions.

For clarity of argumentation, we only contrast the situation with regard to two different temperature regimes (‘cold’ and ‘warm’) but the model applies to any gradual shift in temperature conditions. We define two net-energy intake rates $-b_{C}$ applies to inactive (pre-emergent) bees kept under cold conditions and $-b_{W}$ applies to bees kept under warm conditions. $b_{C}\text{ and }b_{W}$ (or at least the difference between the two) are assumed to be constant throughout the winter period. According to assumption (1.) defined above we specify ${-b}_{W}<{-b}_{C}<0$, i.e. inactive bees loose energy at a greater rate if ambient temperatures are warm. The two intake rates are shown as the two blue and red horizontal lines in Figure 4.

Assumption (2.) is accounted for in the model by letting the net-energy intake rate of an active, viz. emerged, bee increase linearly as the season progresses from early spring to late spring and summer, i.e. $b_{act}=-g_{0}+\alpha\cdot t$ where $\alpha$ specifies the daily increase in net-energy intake rate for active bees. We assume that $-g_{0}<{-b}_{W}$, i.e. that in mid-winter an active (foraging) bee would lose energy at a greater rate than any inactive bee as there are no resources available at this time. The relationship is indicated by the yellow line in Figure 4.

The two simple assumptions above directly define the optimal moment of emergence in spring; any bee should emerge at just that moment/date ($t_{C}$ respectively $t_{W}$) when for the first time the (expected) net energy intake rate of becoming active surpasses the net-energy intake rate when remaining inactive, that is when

$\alpha\cdot t-g_{0}\geq-b_{C}\Rightarrow t_{C}\geq\frac{g_{0}-b_{C}}{\alpha}$ (1a)

and analogously $t_{W}\geq\frac{g_{0}-b_{W}}{\alpha}$. (1b)

Because of ${-b}_{W}<{-b}_{C}$ we conclude that individuals with higher net energy intake rate when inactive should emerge later than those with lower rate, i.e. $t_{C}>t_{W}$ as is observed in the data; the expected time lag in emergence is $\left( b_{W}-b_{C} \right)/\alpha$.

Assumption (3.) indicates that larger bodied bees (or bees that are fitter for other reasons) are more efficient foragers than smaller bees. We account for this effect in the model simply by shifting the yellow line in figure 4, i.e. by assuming a smaller value for $g_{0}$ for large bodied bees (a similar effect would also emerge if they were to expect a larger $\alpha$), and inversely for small bodied bees a larger value for $g_{0}$. From equations (1) we can conclude that reducing $g_{0}$ will result in moving the optimal time of emergence forward as was observed in the data.

A further question is whether the later emerging bees exposed to cooler temperatures will have consumed more or less fat at the time of emergence than those bees that emerged earlier but were exposed to higher temperatures and thus had had lower net energy intake rate in the winter.

The earlier emerging bees (those raised in warm winter conditions) will have lost more (or at least as much) energy at the time of emergence than the later emerging bees kept under cold conditions if

$b_{W}\cdot t_{W}\geq b_{C}\cdot t_{C}$ or $b_{W}\frac{g_{0}-b_{W}}{\alpha}\geq b_{C}\frac{g_{0}-b_{C}}{\alpha}$ (2)

rearranged to

$g_{0}\left( b_{W}-b_{C} \right)\geq b_{W}^{2}-b_{C}^{2}=\left( b_{W}+b_{C} \right)\cdot\left( b_{W}-b_{C} \right)$

and thus

$g_{0}\geq b_{C}+b_{W}$ (3)

we can conclude that the slope parameter $\alpha$ does not affect this inequality.

Whether inequality (3) is true or not thus simply depends on whether $g_{0}$ is smaller or larger than $b_{C}+b_{W}$. Note that $g_{0}$ should not be interpreted as a true net energy intake rate that is valid at $t_{0}$ – it is indeed unlikely that the net intake rate of an active bee would be the lowest just at the onset of winter. Instead, $g_{0}$ is a fictive intersection point that defines the length of the period before the earlier emerging bee become active in relation to the time interval until the later emerging bees become active also.

Both,$b_{C}$ and $b_{W}$ must by definition be smaller than$g_{0}$ but this does not necessarily hold for the sum of both. We can conclude that if $b_{W}$ were close to $g_{0}$ (that is the bees emerge after a brief overwintering time), $b_{C}$ needs to be very small to fulfill the condition. However, it is unreasonable to assume that bees could find food resource in the middle of an even mild winter under mid-European conditions (this would be the implication of $b_{W}$ being close to $g_{0}$) and we thus conclude that eq. (3) should typically hold and consequently that bees kept under warm conditions should burn more fat reserves until the moment of emergence than the later emerging bees kept under cold conditions.
